# Association between Common Polymorphism near the *MC4R* Gene and Obesity Risk: A Systematic Review and Meta-Analysis

**DOI:** 10.1371/journal.pone.0045731

**Published:** 2012-09-25

**Authors:** Bo Xi, Giriraj R. Chandak, Yue Shen, Qijuan Wang, Donghao Zhou

**Affiliations:** 1 Department of Maternal and Child Health Care, School of Public Health, Shandong University, Jinan, Shandong, People's Republic of China; 2 Centre for Cellular and Molecular Biology, Council of Scientific and Industrial Research, Hyderabad, Andhra Pradesh, India; 3 Department of Epidemiology, Capital Institute of Pediatrics, Beijing, People's Republic of China; 4 Department of Endocrinology, Linyi People's Hospital, Linyi, Shandong, People's Republic of China; Sanjay Gandhi Medical Institute, India

## Abstract

**Background:**

Genome-wide association studies on Europeans have shown that two polymorphisms (rs17782313, rs12970134) near the melanocortin 4 receptor (*MC4R*) gene were associated with increased risk of obesity. Subsequently studies among different ethnic populations have shown mixed results with some confirming and others showing inconsistent results, especially among East Asians and Africans. We performed a comprehensive meta-analysis of various studies from different ethnic populations to assess the association of the *MC4R* polymorphism with obesity risk.

**Methods:**

We retrieved all published literature that investigated association of MC4R variants with obesity from PubMed and Embase. Pooled odds ratio (OR) with 95% confidence interval (CI) was calculated using fixed- or random-effects model.

**Results:**

A total of 61 studies (80,957 cases/220,223 controls) for rs17782313 polymorphism (or proxy) were included in the meta-analysis. The results suggested that rs17782313 polymorphism was significantly associated with obesity risk (OR = 1.18, 95%CI = 1.15–1.21, *p*<0.001). Similar trends were observed among subgroups of Europeans and East Asians, adults and children, studies with high quality score, and for each five *MC4R* polymorphisms independently.

**Conclusions:**

The present meta-analysis confirms the significant association of *MC4R* polymorphism with risk of obesity. Further studies should be conducted to identify the causal variant and the underlying mechanisms of the identified association.

## Introduction

Obesity is a major health issue worldwide [1]. According to the World Health Organization, over 400 million people across the globe are obese. Moreover, a number of evidences have established that obesity is associated with increased risk of hypertension, type 2 diabetes and cardiovascular disease [2].

Obesity is a complex disease resulting from genetic and environmental factors, and their interaction [3]. Recently, identification of genetic factors contributing to obesity has been a hot topic. In 2007, the fat mass and obesity associated (*FTO*) gene was identified as the first gene for common obesity by the genome-wide association study (GWAS) [4]. Subsequently, this significant association of *FTO* with obesity has been further replicated in other independent populations [5], [6]. In 2008, the melanocortin 4 receptor (*MC4R*) gene was reported as the second association signal for common obesity by the GWAS [7]. The rs17782313 polymorphism near the *MC4R* gene was found to be associated with obesity among both European adults [odds ratio (OR) = 1.12, 95% confidence interval (CI) = 1.08–1.16, *p* = 5.2×10^−9^)] and children (OR = 1.30, 95%CI: 1.20–1.41, *p* = 8.0×10^−11^) [7]. Another polymorphism (rs12970134) near the *MC4R* gene was also suggested to increase the risk of obesity among Europeans (OR = 1.12, 95%CI 1.06–1.17, *p* = 9.9×10^−16^) [8]. Subsequently, many studies have investigated the association among different ethnic populations [9]–[27]. In addition, other polymorphisms including rs571312 [27]–[31], rs17700144 [32] and rs4450508 [13], [27], which are in high linkage disequilibrium (LD) with rs17782313 or rs12970134 polymorphism, have also been investigated. However, the results have been inconsistent, especially among East Asians and Africans. Although most studies showed significant association, the studies by Hotta et al [9], Tabara et al [10], Liem et al [23], Ng et al [26], and Grant et al [27] revealed non-significant association. The discrepancy might be due to the modest effect of the polymorphism, the limited statistical power for the individual studies with small sample sizes, and the differences in genetic and environmental backgrounds of the studied populations.

Meta-analysis is a useful statistical tool to pool data from individual studies, thereby increasing the statistical power and the precision of effect estimates. In this study, we only focused on obesity rather than the underlying quantitative traits (body mass index (BMI) etc.) since the data provided by the original publications were not uniform (e.g., mean with standard deviation, mean with 95%CI, or beta with 95% CI). Then, we performed a meta-analysis to assess the association between rs17782313 polymorphism near the *MC4R* gene and obesity risk across different ethnic populations.

## Materials and Methods

### Literature and search strategy

We searched the PubMed and Embase databases from 2008 to 2012 since rs17782313 polymorphism in *MC4R* and its association with obesity was firstly reported in 2008. The search strategy to identify all possible studies involved the use of the following key words: (melanocortin 4 receptor or MC4R) and (polymorphism or variant or variation) and obesity. The publication language was restricted to English. The reference lists of retrieved articles were hand-searched. If more than one article were published using the same case series, only the study with largest sample size was included. The literature search was updated on August 1, 2012.

### Inclusion criteria and data extraction

A study was included in the meta-analysis only if it met all the following inclusion criteria: (1) it evaluates the association of any of the *MC4R* polymorphisms (rs17782313, rs12970134, rs571312, rs17700144 and rs4450508) with obesity; (2) uses case-control or cohort design; and (3) provides OR with 95%CI under an additive model or sufficient data for calculation of these estimates. The following information was extracted from each study: (1) name of the first author; (2) year of publication; (3) country of origin; (4) ethnicity of studied population; (5) number of cases and controls; (6) OR with 95%CI under an additive model; (7) covariates adjustment; and (8) BMI criteria for obese cases and controls. Two authors independently assessed the articles for compliance with the inclusion/exclusion criteria, resolved disagreements through discussion and reached a consistent decision.

### Statistical analysis

The association of *MC4R* polymorphism with obesity was estimated by calculating pooled ORs and 95% CIs under an additive model as well as under dominant, recessive and allelic models. The significance of ORs was determined by *Z* test (*p*<0.05 was considered statistically significant). Q test was performed to test the between-study heterogeneity. A random- (DerSimonian-Laird method [33]) or fixed- (Mantel-Haenszel method [34]) effects model was used to calculate pooled effect estimates in the presence (*p*< = 0.10) or absence (*p*>0.10) of heterogeneity, respectively. The included studies were scored based on the criteria selected from published recommendations on the evaluation of the quality of genetic association studies [35]. In addition, we applied “Venice criteria” [36] to assess the credibility of the cumulative evidence of the meta-analyses under all four genetic models. Subgroup analyses were performed by ethnicity (European vs. East Asian vs. African), population (adults (>18 years) vs. children (≤18 years)), the quality score (≥8 vs. <8), and type of polymorphism (rs17782313 vs. rs12970134 vs. rs571312 vs. rs17700144 vs. rs4450508). Publication bias was assessed by Begg's test [37] (*p*<0.05 was considered statistically significant). To evaluate the stability of the results, sensitivity analysis was performed by removing one study at a time. Data analysis was performed using STATA version 11 (StataCorp LP, College Station, TX, USA).

## Results

### Characteristics of the studies

The literature search identified a total of 197 potential relevant articles. Of these, 113 were excluded after reading the title or abstract because of obvious irrelevance. In addition, 7 articles were excluded since they were reviews; one article was excluded because it examined gene-environment interaction; 10 articles were excluded as they assessed the association between *MC4R* gene polymorphism and type 2 diabetes, metabolic syndrome, stroke, polycystic ovary syndrome, or cancer; 4 articles were excluded because they investigated the association between *MC4R* gene polymorphism and dietary intake; 11 articles were excluded as they assessed the associations between other polymorphisms (e.g. V103I (rs2229616) or I251L (rs52820871), which is not in LD with rs17782313 or rs12970134) and obesity; 19 articles were excluded since they investigated the association between *MC4R* gene variants and obesity-related traits, e.g. BMI, waist circumference, waist-to-hip ratio and fat mass percentage; one article was excluded because it included obese subjects also afflicted with polycystic ovary syndrome. Finally, 31 articles met all the primary inclusion criteria. However, two articles were further excluded because they were family-based [38], [39]; one article was excluded because the genotype distribution of rs12970134 was not in Hardy-Weinberg equilibrium in control subjects [40]; one article was excluded because it did not provide sufficient data for calculation OR with 95%CI of rs17782313 [41]; one article was excluded because it was a duplicated publication [42]. Details of the reasons for excluding various studies are summarized in Table S1. In addition, since more than one studies were contained in the articles by Loos et al. [7], Cauchi et al. [11], Meyre et al. [14], Speliotes et al. [30], and Scherag et al. [32], these studies were considered as separate studies in the subsequent data analysis.

**Figure 1:**
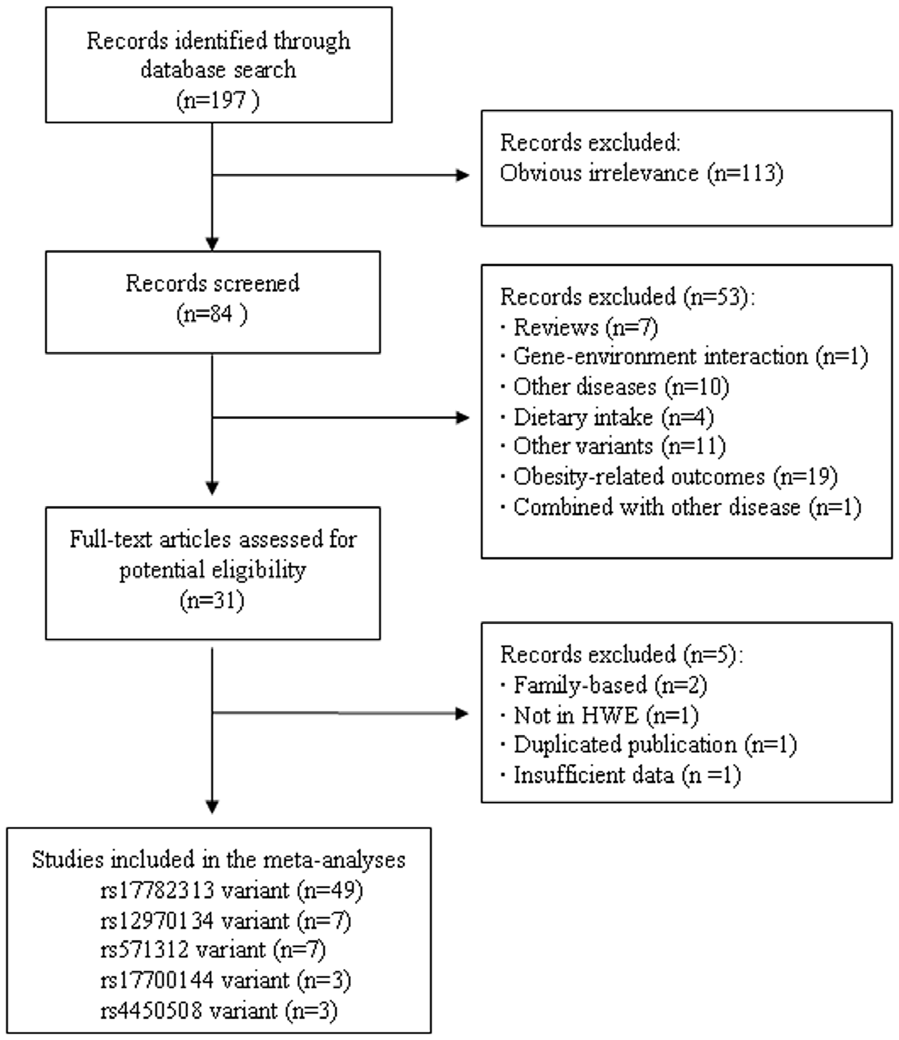
Flow chart of meta-analysis for exclusion/inclusion of studies.

Therefore, 49 studies (48,413cases and 134,392 controls) for rs17782313 polymorphism [7], [9]–[25], 7 studies (19,238 cases and 31,913 controls) for rs12970134 polymorphism [8], [9], [13], [25]–[27], 7 studies (18,060 case and 59,573 controls) for rs571312 polymorphism [27]–[31], 3 studies (2,880 cases and 7,880 controls) for rs17700144 polymorphism [32], and 3 studies (5,609 cases and 11,319 controls) for rs4450508 polymorphism [13], [27] were included in the final meta-analysis. The rs17782313 is used in the data analysis since it is in high linkage disequilibrium with rs12970134 (D′ = 0.95, r^2^ = 0.82 in CEU); D′ = 0.902, r^2^ = 0.813 in CHB; D′ = 0.911, r^2^ = 0.662 in JPT, with rs571312 (D′ = 1, r^2^ = 1 in CEU; D′ = 1, r^2^ = 1 in CHB; D′ = 1, r^2^ = 0.93 in JPT), with rs17700144 (D′ = 1, r^2^ = 0.817 in CEU; D′ = 1, r^2^ = 0.658 in CHB; D′ = 1, r^2^ = 0.689 in JPT), with rs4450508 (D′ = 1, r^2^ = 0.524 in CEU; D′ = 0.574, r^2^ = 0.30 in CHB; D′ = 0.904, r^2^ = 0.599 in JPT). In addition, for the overall meta-analysis, if one study contained more than one polymorphism, only one polymorphism was selected (rs17782313 was chosen for three studies by Hotta et al [9], Zobel et al [13] and Vogel et al [25], while, for two studies by Grant et al [27], data on rs12970134 polymorphism were utilized). Thus, overall data for rs17782313 polymorphism (or its proxy) on 301,180 individuals comprising 80,957 cases and 220,223 controls from 61 studies were included in the meta-analysis of the association under an additive model [7]–[32]. Only 13 studies (22,771 cases and 77,483 controls) provided the genotype frequencies in obese individuals (cases) and normal weight subjects (controls), which were used to calculate the estimates under three other genetic models (dominant, recessive and allelic models). A flow chart describing the process of study inclusion/exclusion is displayed as Figure 1. The characteristics of the included studies are listed in Table S2.

### Meta-analysis results

**Figure 2:**
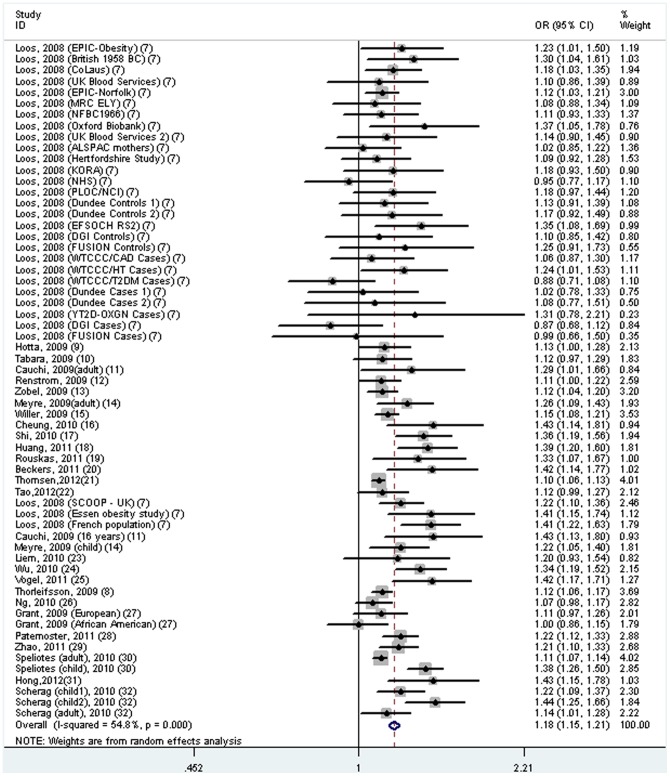
Meta-analysis of the association between rs17782313 polymorphism (or proxy) near the *MC4R* gene and obesity risk under an additive genetic model.

The overall result showed that rs17782313 polymorphism (or its proxy) was significantly associated with obesity risk under an additive model (OR = 1.18, 95%CI 1.15–1.21, *p*<0.001; Table 1 and Figure 2), with evidence of between-study heterogeneity (*I* ^2^ = 54.8%, *p*<0.001). In the stratified subgroups by ethnicity, the effect sizes were significant among both Europeans (OR = 1.18, 95%CI 1.15–1.21, *I* ^2^ = 50.0%, *p* for heterogeneity<0.001) and East Asians (OR = 1.24, 95%CI 1.14–1.34, *I* ^2^ = 67.4%, *p* for heterogeneity = 0.002), but not among Africans (OR = 1.00, 95%CI 0.86–1.16). In addition, there was significant association among both in adults (OR = 1.15, 95%CI 1.12–1.17, *I* ^2^ = 32.9%, *p* for heterogeneity = 0.018) and in children (OR = 1.26, 95%CI 1.19–1.33, *I* ^2^ = 56.8%, *p* for heterogeneity = 0.003). The significant association of rs17782313 (or its proxy) with obesity risk remained even on restricting the analysis to studies with high quality (OR = 1.18, 95%CI = 1.15–1.22, *p* for heterogeneity<0.001). Furthermore, we also performed a subgroup analysis based on different polymorphisms. The results showed that all five polymorphisms were significantly associated with obesity risk (rs17782313: OR = 1.18, 95%CI = 1.15–1.22, *I* ^2^ = 44.2%, *p* for heterogeneity<0.001; rs12970134: OR = 1.12, 95%CI = 1.08–1.15, *I* ^2^ = 12.1%, *p* for heterogeneity = 0.337; rs571312: OR = 1.19, 95%CI = 1.10–1.29, *I* ^2^ = 83.4%, *p* for heterogeneity<0.001; rs17700144: OR = 1.25, 95%CI = 1.10–1.42, *I* ^2^ = 68.2%, *p* for heterogeneity = 0.043; rs4450508: OR = 1.06, 95%CI = 1.01–1.12, *I* ^2^ = 0.0%, *p* for heterogeneity = 0.785) (Table 1).

**Table 1:** Meta-analysis of association between *MC4R* polymorphism and obesity risk under an additive model.

	No. of studies (cases/controls)	OR (95%CI)	*P*_Z-test_	*I* ^2^ (%)	*P*_H_
All^a^	61 (80,957/220,223)	1.18 (1.15–1.21)	<0.001	54.8	<0.001
Ethnicity^a^					
Europeans	51 (70,389/199,765)	1.18 (1.15–1.21)	<0.001	50.0	<0.001
East Asians	9 (9,560/17,743)	1.24 (1.14–1.34)	<0.001	67.4	0.002
Africans	1 (1,008/2,715)	1.00 (0.86–1.16)	1	-	-
Population^a^					
Adults	46 (63,891/182,773)	1.15 (1.12–1.17)	<0.001	32.9	0.018
Children	15 (17,066/37,450)	1.26 (1.19–1.33)	<0.001	56.8	0.003
Quality score^b^					
High (≥8)	41 (58,749/148,557)	1.18 (1.15–1.22)	<0.001	52.9	<0.001
Low (<8)	20 (22,208/71,666)	1.18 (1.12–1.25)	<0.001	58.7	0.001
Polymorphisms^c^					
rs17782313	49 (48,413/134,392)	1.18 (1.15–1.22)	<0.001	44.2	<0.001
rs12970134	7 (19,238/31,913)	1.12 (1.08–1.15)	<0.001	12.1	0.337
rs571312	7 (18,060/59,573)	1.19 (1.10–1.29)	<0.001	83.4	<0.001
rs17700144	3 (2,880/7,880)	1.25 (1.10–1.42)	<0.001	68.2	0.043
rs4450508	3 (5,609/11,319)	1.06 (1.01–1.12)	0.014	0.0	0.785

*Note*s OR, odds ratio; CI, confidence interval; *P*_Z-test_; *P* value for Z test; *P*_H_, *P* value based on Q test for between-study heterogeneity.

a The rs17782313 polymorphism is used in the data analysis since it is in high linkage disequilibrium with four other SNPs, rs12970134, rs571312, rs17700144 and rs4450508 (D′ ranging from 0.90 to 1.00, r^2^ ranging from 0.60 to 0.93 in CEU, CHB and JPT populations).

b See the Methods section.

c There are five studies containing data on more than one *MC4R* polymorphisms.

We also observed significant association of rs17782313 variant (or its proxy) under a dominant model (OR = 1.26, 95%CI = 1.18–1.34, *I* ^2^ = 58.4%, *p* for heterogeneity = 0.004, Figure S1), a recessive model (OR = 1.41, 95% CI = 1.23–1.63, *p* for heterogeneity = 0.001, Figure S2) and an allelic model (OR = 1.24, 95%CI = 1.16–1.32, *p* for heterogeneity<0.001, Figure S3).

Based on the Venice criteria, results under all four genetic models were graded as “A”, “B” and “A” for “amount of evidence”, “replication consistency” and “protection from bias”, repetitively. These results suggested that there was moderate evidence of the association between rs17782313 polymorphism and obesity risk.

### Sensitivity analysis and Publication bias

Sensitivity analysis was performed by excluding one study at a time. The results confirmed the significant association between rs17782313 polymorphism and obesity risk irrespective of the genetic model used for association analysis (Tables S3,S4,S5,S6). Based on the Egger's test, we did not detect any publication bias for rs17782313 polymorphism under an additive model (*p* = 0.695), a dominant model (*p* = 0.200), a recessive model (*p* = 0.300), and an allelic model (*p* = 0.360).

## Discussion

To our knowledge, this is the first meta-analysis investigating the association between *MC4R* polymorphism and susceptibility to obesity across different ethnic populations. The results established that rs17782313 polymorphism near *MC4R* was significantly associated with the increased risk of obesity and similar trends were found among subgroups of Europeans and East Asians, adults and children, studies with high quality, and for each of the five polymorphisms investigated (rs17782313, rs12970134, rs571312, rs17700144, rs4450508).

Although previous studies have reported several rare *MC4R* mutations in the development of extreme and early-onset obesity, recent publications have identified several common genetic polymorphisms near the *MC4R* gene contributing to the common obesity [43]. Two meta-analyses based on candidate gene studies have indicated that two non-synonymous polymorphisms (the V103I and the I251L) have a ∼20% and ∼50% reduced risk of obesity, respectively [44], [45]. In 2008 and 2009, two GWAS identified two new common polymorphisms (rs17782313 and rs12970134), which were associated with risk of obesity among European populations [7], [8]. However, subsequent studies revealed inconsistent results, especially among East Asians and Africans. The present meta-analysis involving a significantly large sample size confirmed the significant association between rs17782313 polymorphism and obesity risk.

Meta-analysis of genetic association studies is usually fraught with the problem of heterogeneity between them [46]. We found significant between-study heterogeneity in the association of rs17782313 variant with obesity risk. Therefore, subgroup analyses based on ethnicity, studied populations, quality scores, and type of polymorphism were performed to explore the source of heterogeneity. However, the between-study heterogeneity persisted in some subgroups suggesting the presence of other unknown confounding factors.

It is possible that the effect sizes of genetic factors predisposing to human diseases are different across various ethnic populations [47]. As is known, the minor allele frequency of rs17782313 polymorphism is only 0.185 in Chinese, but it is 0.265 and 0.314 in Europeans and Africans, respectively (HapMap database). However, the effect size of the polymorphism on obesity was very similar among Europeans and East Asians, while there was no association among Africans.

The effect size of common *MC4R* polymorphism on obesity in children in this meta-analysis (OR = 1.26, 95%CI = 1.19–1.33) was similar with the initial observation in European children (OR = 1.30, 95%CI = 1.20–1.41) [7], but was significantly larger than that in adults in our study (OR = 1.15, 95%CI = 1.12–1.17) (since the 95%CIs of the former and latter ORs did not overlap).

The MC4R is a 332-amino acid protein encoded by a single exon on chromosome 18q22. Evidences have suggested that like *FTO* gene, *MC4R* gene is highly expressed in the central nervous system which regulates the energy metabolism [15]. Several studies have reported that the polymorphisms near the *MC4R* gene play important roles in the modulation of food intake and choice, but not energy expenditure [48]; however, others could not replicate the association with dietary factors [49]. Therefore, further studies are necessary to identify the biological pathways through which the *MC4R* polymorphisms increase obesity susceptibility.

The current meta-analysis has two strengths. First, we used the OR with 95%CI (under an additive model) after covariate adjustment from individual study to calculate the pooled OR, which increased the accuracy of effect estimate. Second, more than 300,000 subjects were included in the meta-analysis, which greatly improved the statistical power. However, several limitations should also be noted. First, different studies used different cut-offs for obesity, which may influence the overall result. However, within each specific ethnic group (European, East Asian or African), the cut-offs were similar. We tried to overcome this shortcoming by performing subgroup analysis by ethnicity, which then indirectly considered the differences of obesity criteria. Second, there was only 1 study in subjects of African ancestry (African Americans), which did not show any effect of *MC4R* variants on risk of obesity. Further studies are required to replicate the association in Africans. Third, the effect of *MC4R* polymorphism on obesity related traits (e.g. BMI, waist circumference, fat mass percentage) were not assessed in the meta-analysis since the data provided by the original publications were not uniform, i.e., several studies provided mean and standard deviation (or 95%CI) across each genotype, while other studies provided beta and 95% CI, which impeded the further data analysis. Indeed, the initial GWAS with ∼14,000 subjects (Indian Asians and Europeans) [50] and two GWAS in East Asians (∼150,000) [51], [52] have confirmed the significant association between *MC4R* polymorphism and BMI, although the association with obesity risk were not addressed among these three GWAS.

## Conclusions

This large meta-analysis confirmed the significant association of rs17782313 polymorphism near the *MC4R* gene with susceptibility to common obesity. Further studies should be conducted to identify the causal variant and the underlying mechanisms of the identified association.

## Supporting Information

Table S1 Details of reasons for exclusion of studies from meta-analysis.
(DOC)

Table S2 Characteristics of studies included in the meta-analysis.
(DOC)

Table S3 Sensitivity analysis under an additive model.
(DOC)

Table S4 Sensitivity analysis under a dominant model.
(DOC)

Table S5 Sensitivity analysis under a recessive model.
(DOC)

Table S6 Sensitivity analysis under an allelic model.
(DOC)

Figure S1 Meta-analysis of the association between rs17782313 polymorphism (or proxy) near the *MC4R* gene and obesity risk under a dominant genetic mod.
(TIF)

Figure S2 Meta-analysis of the association between rs17782313 polymorphism (or proxy) near the *MC4R* gene and obesity risk under a recessive genetic model.
(TIF)

Figure S3 Meta-analysis of the association between rs17782313 polymorphism (or proxy) near the *MC4R* gene and obesity risk under a allelic genetic model.
(TIF)
