# Supplementary material for: Association between Common Polymorphism near the MC4R Gene and Obesity Risk: A Systematic Review and Meta-Analysis
Source: PLoS One. 2012 Sep 25;7(9):e45731. doi: 10.1371/journal.pone.0045731 (PMC3458070; doi:10.1371/journal.pone.0045731)
Supplement: Table S1 — Details of reasons for exclusion of studies from meta-analysis. (DOC) [file pone.0045731.s001.doc]

Supplementary table 1: Details of reasons for exclusion of studies from meta-analysis

| References | Reason for exclusion |
| --- | --- |
| 1 – 7 | Reviews |
| 8 | Gene-environment interaction |
| 9 -18 | Other disease |
| 19 -22 | Dietary intake |
| 23 -33 | Other variants |
| 34-52 | Obesity-related outcomes |
| 53 | Combined with other disease |
| 54, 55 | Family-based |
| 56 | Data not in HWE |
| 57 | Duplicated publication |
| 58 | Insufficient data |

**References**

1. Ramachandrappa S, Farooqi IS (2011) Genetic approaches to understanding human obesity. J Clin Invest 121:2080-2086.
2. Loos RJ (2011) The genetic epidemiology of melanocortin 4 receptor variants. Eur J Pharmacol 660:156-164.
3. Razquin C, Marti A, Martinez JA (2011) Evidences on three relevant obesogenes: MC4R, FTO and PPARγ. Approaches for personalized nutrition. Mol Nutr Food Res 55:136-149.
4. Hetherington MM, Cecil JE (2010) Gene-environment interactions in obesity. Forum Nutr 63:195-203.
5. Beckers S, Zegers D, Van Gaal LF, Van Hul W (2009) The role of the leptin-melanocortin signalling pathway in the control of food intake. Crit Rev Eukaryot Gene Expr 19:267-287.
6. Dina C (2008) New insights into the genetics of body weight. Curr Opin Clin Nutr Metab Care 11:378-384.
7. Hinney A, Hebebrand J (2008) Polygenic obesity in humans. Obes Facts 1:35-42.
8. Xi B, Wang C, Wu L, Zhang M, Shen Y, et al (2011) Influence of physical inactivity on associations between single nucleotide polymorphisms and genetic predisposition to childhood obesity. Am J Epidemiol 173:1256-1262.
9. Delahanty RJ, Beeghly-Fadiel A, Xiang YB, Long J, Cai Q, et al. (2011) Association of obesity-related genetic variants with endometrial cancer risk: a report from the Shanghai Endometrial Cancer Genetics Study. Am J Epidemiol 174:1115-1126.
10. Winter Y, Back T, Scherag A, Linseisen J, Rohrmann S, et al. (2011) Evaluation of the obesity genes FTO and MC4R and the type 2 diabetes mellitus gene TCF7L2 for contribution to stroke risk: The Mannheim-Heidelberg Stroke Study. Obes Facts 4:290-296.
11. Janipalli CS, Kumar MV, Vinay DG, Sandeep MN, Bhaskar S, et al. (2012) Analysis of 32 common susceptibility genetic variants and their combined effect in predicting risk of Type 2 diabetes and related traits in Indians. Diabet Med 29:121-127.
12. Hotta K, Kitamoto T, Kitamoto A, Mizusawa S, Matsuo T, et al. (2011) Association of variations in the FTO, SCG3 and MTMR9 genes with metabolic syndrome in a Japanese population. J Hum Genet 56:647-651.
13. Kusinska R, Górniak P, Pastorczak A, Fendler W, Potemski P, et al. (2012) Influence of genomic variation in FTO at 16q12.2, MC4R at 18q22 and NRXN3 at 14q31 genes on breast cancer risk. Mol Biol Rep 39:2915-2919.
14. Takeuchi F, Yamamoto K, Katsuya T, Nabika T, Sugiyama T, et al. (2011) Association of genetic variants for susceptibility to obesity with type 2 diabetes in Japanese individuals. Diabetologia 54:1350-1359.
15. Lurie G, Gaudet MM, Spurdle AB, Carney ME, Wilkens LR, et al. (2011) The obesity-associated polymorphisms FTO rs9939609 and MC4R rs17782313 and endometrial cancer risk in non-Hispanic white women. PLoS One 6(2):e16756.
16. Ranjith N, Pegoraro RJ, Shanmugam R (2011) Obesity-associated genetic variants in young Asian Indians with the metabolic syndrome and myocardial infarction. Cardiovasc J Afr 22:25-30.
17. Cheung CY, Tso AW, Cheung BM, Xu A, Ong KL, et al. (2011) Genetic variants associated with persistent central obesity and the metabolic syndrome in a 12-year longitudinal study. Eur J Endocrinol 164:381-388.
18. Tan S, Scherag A, Janssen OE, Hahn S, Lahner H, et al.(2010) Large effects on body mass index and insulin resistance of fat mass and obesity associated gene (FTO) variants in patients with polycystic ovary syndrome (PCOS).BMC Med Genet 11:12.
19. Hasselbalch AL, Angquist L, Christiansen L, Heitmann BL, Kyvik KO, et al. (2010) A variant in the fat mass and obesity-associated gene (FTO) and variants near the melanocortin-4 receptor gene (MC4R) do not influence dietary intake. J Nutr 140:831-834.
20. Cole SA, Butte NF, Voruganti VS, Cai G, Haack K, et al. (2010) Evidence that multiple genetic variants of MC4R play a functional role in the regulation of energy expenditure and appetite in Hispanic children. Am J Clin Nutr 91:191-199.
21. Stutzmann F, Cauchi S, Durand E, Calvacanti-Proença C, Pigeyre M, et al. (2009) Common genetic variation near MC4R is associated with eating behaviour patterns in European populations. Int J Obes (Lond) 33:373-378.
22. Qi L, Kraft P, Hunter DJ, Hu FB. (2008) The common obesity variant near MC4R gene is associated with higher intakes of total energy and dietary fat, weight change and diabetes risk in women. Hum Mol Genet 17:3502-3508.
23. Nowacka-Woszuk J, Cieslak J, Skowronska B, Majewska KA, Stankiewicz W, et al. (2011) Missense mutations and polymorphisms of the MC4R gene in Polish obese children and adolescents in relation to the relative body mass index. J Appl Genet 52:319-323.
24. van den Berg L, van Beekum O, Heutink P, Felius BA, van de Heijning MP, et al. (2011) Melanocortin-4 receptor gene mutations in a Dutch cohort of obese children.Obesity (Silver Spring) 19:604-611.
25. Demiralp DO, Berberoglu M, Akar N. (2011) Melanocortin-4 receptor polymorphisms in Turkish pediatric obese patients. Clin Appl Thromb Hemost 17:70-74.
26. Kalnina I, Kapa I, Pirags V, Ignatovica V, Schiöth HB, et al. (2009) Association between a rare SNP in the second intron of human Agouti related protein gene and increased BMI.BMC Med Genet 10:63.
27. Santoro N, Cirillo G, Xiang Z, Tanas R, Greggio N,et al. (2009) Prevalence of pathogenetic MC4R mutations in Italian children with early onset obesity, tall stature and familial history of obesity. BMC Med Genet 10:25.
28. Roth CL, Ludwig M, Woelfle J, Fan ZC, Brumm H, et al. (2009) A novel melanocortin-4 receptor gene mutation in a female patient with severe childhood obesity. Endocrine 36:52-59.
29. Yurtcu E, Yilmaz A, Ozkurt Z, Kolukisa E, Yilmaz M, et al. (2009) Melanocortin-4 receptor gene polymorphisms in obese patients. Biochem Genet 47:295-300.
30. Tenesa A, Campbell H, Theodoratou E, Dunlop L, Cetnarskyj R, et al. (2009) Common genetic variants at the MC4R locus are associated with obesity, but not with dietary energy intake or colorectal cancer in the Scottish population. Int J Obes (Lond) 33:284-288.
31. Pichler M, Kollerits B, Heid IM, Hunt SC, Adams TD, et al. (2008) Association of the melanocortin-4 receptor V103I polymorphism with dietary intake in severely obese persons. Am J Clin Nutr 88:797-800.
32. Valli-Jaakola K, Suviolahti E, Schalin-Jäntti C, Ripatti S, Silander K, et al. (2008) Further evidence for the role of ENPP1 in obesity: association with morbid obesity in Finns. Obesity (Silver Spring) 16:2113-2119.
33. Kring SI, Larsen LH, Holst C, Toubro S, Hansen T, et al. (2008) Genotype-phenotype associations in obesity dependent on definition of the obesity phenotype. Obes Facts 1:138-145.
34. Okada Y, Kubo M, Ohmiya H, Takahashi A, Kumasaka N, et al. (2012) Common variants at CDKAL1 and KLF9 are associated with body mass index in east Asian populations. Nat Genet 44:302-306.
35. Wen W, Cho YS, Zheng W, Dorajoo R, Kato N, et al. (2012) Meta-analysis identifies common variants associated with body mass index in east Asians. Nat Genet 44:307-311.
36. Klimentidis YC, Chen GB, López-Alarcón M, Harris JJ, Duarte CW, et al. (2011) Associations of obesity genes with obesity-related outcomes in multiethnic children. Arch Med Res 42:509-514.
37. Coenen KR, Karp SM, Gesell SB, Dietrich MS, Morgan TM, et al. (2011) Genetic risk score does not correlate with body mass index of Latina women in a clinical trial. Clin Transl Sci 4:323-327.
38. Kuo PH, Kao CF, Chen PY, Chen CH, Tsai YS, et al. (2011) Polymorphisms of INSIG2, MC4R, and LEP are associated with obesity- and metabolic-related traits in schizophrenic patients. J Clin Psychopharmacol 31:705-711.
39. Hunt SC, Hasstedt SJ, Xin Y, Dalley BK, Milash BA, et al. (2011) Polymorphisms in the NPY2R gene show significant associations with BMI that are additive to FTO, MC4R, and NPFFR2 gene effects. Obesity (Silver Spring) 19:2241-2247.
40. Hester JM, Wing MR, Li J, Palmer ND, Xu J, et al. (2012) Implication of European-derived adiposity loci in African Americans. Int J Obes (Lond) 36:465-473.
41. Liu G, Zhu H, Dong Y, Podolsky RH, Treiber FA, et al. (2011) Influence of common variants in FTO and near INSIG2 and MC4R on growth curves for adiposity in African- and European-American youth. Eur J Epidemiol 26:463-473.
42. Dorajoo R, Blakemore AI, Sim X, Ong RT, Ng DP, et al. (2012) Replication of 13 obesity loci among Singaporean Chinese, Malay and Asian-Indian populations. Int J Obes (Lond) 36:159-163.
43. Orkunoglu-Suer FE, Harmon BT, Gordish-Dressman H, Clarkson PM, Thompson PD, et al. (2011) MC4R variant is associated with BMI but not response to resistance training in young females. Obesity (Silver Spring) 19:662-666.
44. Hotta K, Nakamura M, Nakamura T, Matsuo T, Nakata Y, et al. (2010) Polymorphisms in NRXN3, TFAP2B, MSRA, LYPLAL1, FTO and MC4R and their effect on visceral fat area in the Japanese population. J Hum Genet 55:738-742.
45. Holzapfel C, Grallert H, Huth C, Wahl S, Fischer B, et al. (2010) Genes and lifestyle factors in obesity: results from 12,462 subjects from MONICA/KORA. Int J Obes (Lond) 34:1538-1545.
46. Petry CJ, López-Bermejo A, Díaz M, Sebastiani G, Ong KK, et al. (2010) Association between a common variant near MC4R and change in body mass index develops by two weeks of age. Horm Res Paediatr 73:275-280.
47. Liu G, Zhu H, Lagou V, Gutin B, Barbeau P, et al. (2010) Common variants near melanocortin 4 receptor are associated with general and visceral adiposity in European- and African-American youth. J Pediatr 156:598-605.e1.
48. Kring SI, Holst C, Toubro S, Astrup A, Hansen T, et al. (2010) Common variants near MC4R in relation to body fat, body fat distribution, metabolic traits and energy expenditure. Int J Obes (Lond) 34:182-189.
49. Wang D, Ma J, Zhang S, Hinney A, Hebebrand J, et al. (2010) Association of the MC4R V103I polymorphism with obesity: a Chinese case-control study and meta-analysis in 55,195 individuals. Obesity (Silver Spring) 18:573-579.
50. Bauer F, Elbers CC, Adan RA, Loos RJ, Onland-Moret NC, et al. (2009) Obesity genes identified in genome-wide association studies are associated with adiposity measures and potentially with nutrient-specific food preference. Am J Clin Nutr 90:951-959.
51. Been LF, Nath SK, Ralhan SK, Wander GS, Mehra NK, et al. (2010) Replication of association between a common variant near melanocortin-4 receptor gene and obesity-related traits in Asian Sikhs. Obesity (Silver Spring) 18:425-429.
52. Haupt A, Thamer C, Heni M, Tschritter O, Machann J,et al. (2009) Impact of variation near MC4R on whole-body fat distribution, liver fat, and weight loss. Obesity (Silver Spring) 17:1942-1945.
53. Ewens KG, Jones MR, Ankener W, Stewart DR, Urbanek M,et al. (2011) FTO and MC4R gene variants are associated with obesity in polycystic ovary syndrome. PLoS One 6:e16390.
54. Scherag A, Jarick I, Grothe J, Biebermann H, Scherag S, et al. (2010) Investigation of a genome wide association signal for obesity: synthetic association and haplotype analyses at the melanocortin 4 receptor gene locus. PLoS One 5:e13967.
55. Valladares M, Domínguez-Vásquez P, Obregón AM, Weisstaub G, Burrows R, et al. (2010) Melanocortin-4 receptor gene variants in Chilean families: association with childhood obesity and eating behavior. Nutr Neurosci 13:71–78.
56. Huang H, Zeng Z, Zhang L, Liu R, Li X, et al. (2012) Implication of genetic variants near TMEM18, BCDIN3D/FAIM2, and MC4R with coronary artery disease and obesity in Chinese: a angiography-based study. Mol Biol Rep 39: 1739–1744.
57. Li S, Zhao JH, Luan J, Luben RN, Rodwell SA, et al. (2010) Cumulative effects and predictive value of common obesity-susceptibility variants identified by genome-wide association studies. Am J Clin Nutr 91: 184–190.
58. Wang J, Mei H, Chen W, Jiang Y, Sun W, et al. (2012) Study of eight GWAS-identified common variants for association with obesity-related indices in Chinese children at puberty. Int J Obes (Lond) 36, 542-547.
