# Supplementary material for: Association between Common Polymorphism near the MC4R Gene and Obesity Risk: A Systematic Review and Meta-Analysis
Source: PLoS One. 2012 Sep 25;7(9):e45731. doi: 10.1371/journal.pone.0045731 (PMC3458070; doi:10.1371/journal.pone.0045731)
Supplement: Table S2 — Characteristics of studies included in the meta-analysis. (DOC) [file pone.0045731.s002.doc]

**Supplementary table 2: Characteristics of studies included in the meta-analysis**

| **SNP/Study** | **Country** | **Ethnicity** | **No. of cases** | **No. of controls** | **Odds**  **ratio^a^** | **95% confidence**  **interval^a^** | | **Quality**  **score^b^** | **Criteria^c^, BMI, kg/m^2^** | |
| --- | --- | --- | --- | --- | --- | --- | --- | --- | --- | --- |
|  |  |  |  |  |  |  |  |  | Cases | Controls |
| **rs17782313** |  |  |  |  |  |  |  |  |  |  |
| Loos, 2008 (EPIC-Obesity) (7) | UK | European | 380 | 920 | 1.23 | 1.01 | 1.50 | 8 | >=30 | <25 |
| Loos, 2008 (British 1958 BC) (7) | UK | European | 368 | 494 | 1.30 | 1.04 | 1.61 | 8 | >=30 | <25 |
| Loos, 2008 (CoLaus) (7) | Switzerland | European | 889 | 2,685 | 1.18 | 1.03 | 1.35 | 8 | >=30 | <25 |
| Loos, 2008 (UK Blood Services) (7) | UK | European | 139 | 888 | 1.10 | 0.86 | 1.39 | 8 | >=30 | <25 |
| Loos, 2008 (EPIC-Norfolk) (7) | UK | European | 2,346 | 6,555 | 1.12 | 1.03 | 1.21 | 9 | >=30 | <25 |
| Loos, 2008 (MRC ELY) (7) | UK | European | 381 | 598 | 1.08 | 0.88 | 1.34 | 8 | >=30 | <25 |
| Loos, 2008 (NFBC1966) (7) | Finland | European | 473 | 3031 | 1.11 | 0.93 | 1.33 | 8 | >=30 | <25 |
| Loos, 2008 (Oxford Biobank) (7) | UK | European | 200 | 524 | 1.37 | 1.05 | 1.78 | 8 | >=30 | <25 |
| Loos, 2008 (UK Blood Services 2) (7) | UK | European | 268 | 706 | 1.14 | 0.90 | 1.45 | 8 | >=30 | <25 |
| Loos, 2008 (ALSPAC mothers) (7) | UK | European | 347 | 4,929 | 1.02 | 0.85 | 1.22 | 8 | >=30 | <25 |
| Loos, 2008 (Hertfordshire Study) (7) | UK | European | 683 | 881 | 1.09 | 0.92 | 1.28 | 8 | >=30 | <25 |
| Loos, 2008 (KORA) (7) | Germany | European | 356 | 467 | 1.18 | 0.93 | 1.50 | 8 | >=30 | <25 |
| Loos, 2008 (NHS) (7) | UK | European | 293 | 1,334 | 0.95 | 0.77 | 1.17 | 8 | >=30 | <25 |
| Loos, 2008 (PLOC/NCI) (7) | USA | European | 504 | 579 | 1.18 | 0.97 | 1.44 | 8 | >=30 | <25 |
| Loos, 2008 (Dundee Controls 1) (7) | UK | European | 391 | 769 | 1.13 | 0.91 | 1.39 | 8 | >=30 | <25 |
| Loos, 2008 (Dundee Controls 2) (7) | UK | European | 319 | 530 | 1.17 | 0.92 | 1.49 | 8 | >=30 | <25 |
| Loos, 2008 (EFSOCH RS2) (7) | UK | European | 322 | 866 | 1.35 | 1.08 | 1.69 | 8 | >=30 | <25 |
| Loos, 2008 (DGI Controls) (7) | USA | European | 249 | 529 | 1.10 | 0.85 | 1.42 | 8 | >=30 | <25 |
| Loos, 2008 (FUSION Controls) (7) | Finland and USA | European | 267 | 430 | 1.25 | 0.91 | 1.73 | 8 | >=30 | <25 |
| Loos, 2008 (WTCCC/CAD Cases) (7) | UK | European | 560 | 492 | 1.06 | 0.87 | 1.30 | 7 | >=30 | <25 |
| Loos, 2008 (WTCCC/HT Cases) (7) | UK | European | 539 | 486 | 1.24 | 1.01 | 1.53 | 7 | >=30 | <25 |
| Loos, 2008 (WTCCC/T2DM Cases) (7) | UK | European | 1,022 | 257 | 0.88 | 0.71 | 1.08 | 7 | >=30 | <25 |
| Loos, 2008 (Dundee Cases 1) (7) | UK | European | 1,134 | 209 | 1.02 | 0.78 | 1.33 | 7 | >=30 | <25 |
| Loos, 2008 (Dundee Cases 2) (7) | UK | European | 644 | 123 | 1.08 | 0.77 | 1.51 | 7 | >=30 | <25 |
| Loos, 2008 (YT2D-OXGN Cases) (7) | UK | European | 372 | 62 | 1.31 | 0.78 | 2.21 | 7 | >=30 | <25 |
| Loos, 2008 (DGI Cases) (7) | USA | European | 495 | 323 | 0.87 | 0.68 | 1.12 | 7 | >=30 | <25 |
| Loos, 2008 (FUSION Cases) (7) | Finland and USA | European | 529 | 124 | 0.99 | 0.66 | 1.50 | 7 | >=30 | <25 |
| Hotta, 2009 (9) | Japan | East Asian | 1,129 | 1,733 | 1.13 | 1.00 | 1.28 | 8 | >=30 | <25 |
| Tabara, 2009 (10) | Japan | East Asian | 804 | 2,002 | 1.12 | 0.97 | 1.29 | 7 | >=25 | <25 |
| Cauchi, 2009(adult) (11) | France | European | 213 | 1,047 | 1.29 | 1.01 | 1.66 | 7 | >=30 | <25 |
| Renstrom, 2009 (12) | Sweden | European | 353 | 1,370 | 1.11 | 1.00 | 1.22 | 6 | >=30 | 18.5-25 |
| Zobel, 2009 (13) | Denmark | European | 3,913 | 4,721 | 1.12 | 1.04 | 1.20 | 8 | >=30 | <25 |
| Meyre, 2009(adult) (14) | Switzerland and France | European | 1,171 | 1,114 | 1.26 | 1.09 | 1.43 | 9 | >=30 | <25 |
| Willer, 2009 (15) | UK, Finland, Botnia | European | 5,261 | 14,106 | 1.15 | 1.08 | 1.21 | 9 | >=30 | 18.5-25 |
| Cheung, 2010 (16) | China | East Asian | 464 | 692 | 1.43 | 1.14 | 1.81 | 7 | ≥27.5 | 18.5-23 |
| Shi, 2010 (17) | China | East Asian | 830 | 1,863 | 1.36 | 1.19 | 1.56 | 8 | ≥27.5 | 18.5-23 |
| Huang, 2011 (18) | China | East Asian | 560 | 1,200 | 1.39 | 1.20 | 1.60 | 6 | ≥28 | 18-24 |
| Rouskas, 2011 (19) | Greece | European | 510 | 469 | 1.33 | 1.07 | 1.67 | 5 | >=30 | <25 |
| Beckers, 2011 (20) | Belgium | European | 1,049 | 312 | 1.42 | 1.14 | 1.77 | 5 | >=30 | 18.5-25 |
| Thomsen, 2012 (21) | Denmark | European | 10,555 | 54,257 | 1.1 | 1.06 | 1.13 | 7 | >=30 | <25 |
| Tao, 2012 (22) | China | East Asian | 914 | 3571 | 1.12 | 0.99 | 1.27 | 8 | ≥27.5 | 18.5-23 |
| Loos, 2008 (SCOOP - UK) (7) | UK | European | 1,028 | 5,988 | 1.22 | 1.10 | 1.36 | 9 | >=mean  +3sd | <mean  +3sd |
| Loos, 2008 (Essen obesity study) (7) | Germany | European | 487 | 442 | 1.41 | 1.15 | 1.74 | 8 | >=95th | <95th |
| Loos, 2008 (French population) (7) | France | European | 1,291 | 1,347 | 1.41 | 1.22 | 1.63 | 9 | >=97th | <97th |
| Cauchi, 2009 (16 years) (11) | Finland | European | 252 | 4,062 | 1.43 | 1.13 | 1.80 | 7 | >=97th | 10th-90th |
| Meyre, 2009 (child) (14) | Finland | European | 896 | 1,297 | 1.22 | 1.05 | 1.40 | 9 | >=97th | <90th |
| Liem, 2010 (23) | Netherlands | European | 175 | 985 | 1.20 | 0.93 | 1.54 | 6 | >=95th | <95th |
| Wu, 2010 (24) | China | East Asian | 1,207 | 1,589 | 1.34 | 1.19 | 1.52 | 8 | >=95th | <90th |
| Vogel, 2011 (25) | Germany | European | 881 | 434 | 1.42 | 1.17 | 1.71 | 6 | >=85th | <85th |
|  |  |  |  |  |  |  |  |  |  |  |
| **rs12970134** |  |  |  |  |  |  |  |  |  |  |
| Thorleifsson, 2009 (8) | Iceland, Netherlands, Denmark, USA | European | 8,492 | 13,785 | 1.12 | 1.06 | 1.17 | 9 | >=30 | 18.5-25 |
| Hotta, 2009 (9) | Japan | East Asian | 1,129 | 1,733 | 1.06 | 0.91 | 1.23 | 8 | >=30 | <25 |
| Zobel, 2009 (13) | Denmark | European | 3,886 | 4,694 | 1.15 | 1.08 | 1.23 | 8 | >=30 | <25 |
| Ng, 2010 (26) | China | East Asian | 3,112 | 4,593 | 1.07 | 0.98 | 1.17 | 9 | >=25 | <25 |
| Grant, 2009 (European) (27) | USA | European | 728 | 3,960 | 1.11 | 0.97 | 1.26 | 9 | >=95th | <95th |
| Grant, 2009 (African American) (27) | USA | African American | 1,008 | 2,715 | 1.00 | 0.86 | 1.15 | 9 | >=95th | <95th |
| Vogel, 2011 (25) | Germany | European | 883 | 433 | 1.29 | 1.08 | 1.55 | 6 | >=85th | <85th |
| **rs571312** |  |  |  |  |  |  |  |  |  |  |
| Grant, 2009 (European) (27) | USA | European | 728 | 3,960 | 1.14 | 1.00 | 1.30 | 9 | >=95th | <95th |
| Grant, 2009 (African American) (27) | USA | African American | 1,008 | 2,715 | 1.01 | 0.91 | 1.13 | 9 | >=95th | <95th |
| Paternoster, 2011 (28) | Denmark | European | 2,633 | 2,740 | 1.22 | 1.12 | 1.33 | 8 | >=31 | <31 |
| Zhao, 2011 (29) | USA | European | 1,097 | 2,760 | 1.21 | 1.10 | 1.33 | 7 | >=95th | <50th |
| Speliotes (adult), 2010 (30) | European countries | European | 6,918 | 42,907 | 1.10 | 1.07 | 1.14 | 9 | >=30 | <25 |
| Speliotes (child), 2010 (30) | Germany, France, USA, UK | European | 5,136 | 3,991 | 1.38 | 1.26 | 1.50 | 8 | Extreme obesity | Normal weight |
| Hong, 2012 (31) | China | East Asian | 540 | 500 | 1.43 | 1.15 | 1.78 | 8 | >=30 | <23 |
| **rs17700144** |  |  |  |  |  |  |  |  |  |  |
| Scherag (child1), 2010 (32) | French | European | 1,181 | 1,960 | 1.22 | 1.09 | 1.37 | 9 | >=90th | <90th |
| Scherag (child2), 2010 (32) | Germany | European | 711 | 1,803 | 1.44 | 1.25 | 1.66 | 9 | >=90th | <90th |
| Scherag (adult), 2010 (32) | Germany | European | 988 | 4,117 | 1.14 | 1.01 | 1.28 | 9 | >=30 | <25 |
| **rs4450508** |  |  |  |  |  |  |  |  |  |  |
| Grant, 2009 (European) (27) | USA | European | 728 | 3,960 | 1.10 | 0.98 | 1.24 | 9 | >=95th | <95th |
| Grant, 2009 (African American) (27) | USA | African American | 1,008 | 2,715 | 1.04 | 0.93 | 1.16 | 9 | >=95th | <95th |
| Zobel, 2009 (13) | Denmark | European | 3,873 | 4,644 | 1.06 | 1.00 | 1.13 | 8 | >=30 | <25 |

^a^Under an additive model

^b^See the Methods section

^c^The percentile of BMI values were used to define obese cases and normal weight controls for nearly most studies on children.

Figures in parentheses indicate the reference number in the main manuscript.
