# Supplementary material for: Association between Common Polymorphism near the MC4R Gene and Obesity Risk: A Systematic Review and Meta-Analysis
Source: PLoS One. 2012 Sep 25;7(9):e45731. doi: 10.1371/journal.pone.0045731 (PMC3458070; doi:10.1371/journal.pone.0045731)
Supplement: Table S3 — Sensitivity analysis under an additive model. (DOC) [file pone.0045731.s003.doc]

**Supplementary table 3: Sensitivity analysis under an additive model**

| **Study omitted** | **OR** | **95% CI** | |
| --- | --- | --- | --- |
| Loos, 2008 (EPIC-Obesity) (7) | 1.15 | 1.13 | 1.17 |
| Loos, 2008 (British 1958 BC) (7) | 1.15 | 1.13 | 1.17 |
| Loos, 2008 (CoLaus) (7) | 1.15 | 1.13 | 1.17 |
| Loos, 2008 (UK Blood Services) (7) | 1.15 | 1.13 | 1.17 |
| Loos, 2008 (EPIC-Norfolk) (7) | 1.15 | 1.14 | 1.17 |
| Loos, 2008 (MRC ELY) (7) | 1.15 | 1.13 | 1.17 |
| Loos, 2008 (NFBC1966) (7) | 1.15 | 1.13 | 1.17 |
| Loos, 2008 (Oxford Biobank) (7) | 1.15 | 1.13 | 1.17 |
| Loos, 2008 (UK Blood Services 2) (7) | 1.15 | 1.13 | 1.17 |
| Loos, 2008 (ALSPAC mothers) (7) | 1.15 | 1.14 | 1.17 |
| Loos, 2008 (Hertfordshire Study) (7) | 1.15 | 1.13 | 1.17 |
| Loos, 2008 (KORA) (7) | 1.15 | 1.13 | 1.17 |
| Loos, 2008 (NHS) (7) | 1.15 | 1.14 | 1.17 |
| Loos, 2008 (PLOC/NCI) (7) | 1.15 | 1.13 | 1.17 |
| Loos, 2008 (Dundee Controls 1) (7) | 1.15 | 1.13 | 1.17 |
| Loos, 2008 (Dundee Controls 2) (7) | 1.15 | 1.13 | 1.17 |
| Loos, 2008 (EFSOCH RS2) (7) | 1.15 | 1.13 | 1.17 |
| Loos, 2008 (DGI Controls) (7) | 1.15 | 1.13 | 1.17 |
| Loos, 2008 (FUSION Controls) (7) | 1.15 | 1.13 | 1.17 |
| Loos, 2008 (WTCCC/CAD Cases) (7) | 1.15 | 1.13 | 1.17 |
| Loos, 2008 (WTCCC/HT Cases) (7) | 1.15 | 1.13 | 1.17 |
| Loos, 2008 (WTCCC/T2DM Cases) (7) | 1.15 | 1.14 | 1.17 |
| Loos, 2008 (Dundee Cases 1) (7) | 1.15 | 1.13 | 1.17 |
| Loos, 2008 (Dundee Cases 2) (7) | 1.15 | 1.13 | 1.17 |
| Loos, 2008 (YT2D-OXGN Cases) (7) | 1.15 | 1.13 | 1.17 |
| Loos, 2008 (DGI Cases) (7) | 1.15 | 1.14 | 1.17 |
| Loos, 2008 (FUSION Cases) (7) | 1.15 | 1.13 | 1.17 |
| Hotta, 2009 (9) | 1.15 | 1.13 | 1.17 |
| Tabara, 2009 (10) | 1.15 | 1.13 | 1.17 |
| Cauchi, 2009(adult) (11) | 1.15 | 1.13 | 1.17 |
| Renstrom, 2009 (12) | 1.15 | 1.14 | 1.17 |
| Zobel, 2009 (13) | 1.15 | 1.14 | 1.17 |
| Meyre, 2009(adult) (14) | 1.15 | 1.13 | 1.17 |
| Willer, 2009 (15) | 1.15 | 1.13 | 1.17 |
| Cheung, 2010 (16) | 1.15 | 1.13 | 1.17 |
| Shi, 2010 (17) | 1.15 | 1.13 | 1.16 |
| Huang, 2011 (18) | 1.15 | 1.13 | 1.16 |
| Rouskas, 2011 (19) | 1.15 | 1.13 | 1.17 |
| Beckers, 2011 (20) | 1.15 | 1.13 | 1.17 |
| Thomsen,2012(21) | 1.16 | 1.14 | 1.18 |
| Tao,2012(22) | 1.15 | 1.13 | 1.17 |
| Loos, 2008 (SCOOP - UK) (7) | 1.15 | 1.13 | 1.17 |
| Loos, 2008 (Essen obesity study) (7) | 1.15 | 1.13 | 1.17 |
| Loos, 2008 (French population) (7) | 1.15 | 1.13 | 1.16 |
| Cauchi, 2009 (16 years) (11) | 1.15 | 1.13 | 1.17 |
| Meyre, 2009 (child) (14) | 1.15 | 1.13 | 1.17 |
| Liem, 2010 (23) | 1.15 | 1.13 | 1.17 |
| Wu, 2010 (24) | 1.15 | 1.13 | 1.16 |
| Vogel, 2011 (25) | 1.15 | 1.13 | 1.16 |
| Thorleifsson, 2009 (8) | 1.15 | 1.14 | 1.17 |
| Ng, 2010 (26) | 1.15 | 1.14 | 1.17 |
| Grant, 2009 (European) (27) | 1.15 | 1.13 | 1.17 |
| Grant, 2009 (African American) (27) | 1.15 | 1.14 | 1.17 |
| Paternoster, 2011 (28) | 1.15 | 1.13 | 1.16 |
| Zhao, 2011 (29) | 1.15 | 1.13 | 1.16 |
| Speliotes (adult), 2010 (30) | 1.16 | 1.14 | 1.18 |
| Speliotes (child), 2010 (30) | 1.14 | 1.13 | 1.16 |
| Hong,2012(31) | 1.15 | 1.13 | 1.17 |
| Scherag (child1), 2010 (32) | 1.15 | 1.13 | 1.17 |
| Scherag (child2), 2010 (32) | 1.15 | 1.13 | 1.16 |
| Scherag (adult), 2010 (32) | 1.15 | 1.13 | 1.17 |

OR, odds ratio; CI, confidence interval
