# Supplementary material for: Association between Common Polymorphism near the MC4R Gene and Obesity Risk: A Systematic Review and Meta-Analysis
Source: PLoS One. 2012 Sep 25;7(9):e45731. doi: 10.1371/journal.pone.0045731 (PMC3458070; doi:10.1371/journal.pone.0045731)
Supplement: Table S4 — Sensitivity analysis under a dominant model. (DOC) [file pone.0045731.s004.doc]

**Supplementary table 4: Sensitivity analysis under a dominant model**

| **Study omitted** | **OR** | **95% CI** | |
| --- | --- | --- | --- |
| Hotta, 2009 (9) | 1.27 | 1.18 | 1.36 |
| Tabara, 2009 (10) | 1.27 | 1.19 | 1.36 |
| Cauchi, 2009 (adult) (11) | 1.25 | 1.17 | 1.34 |
| Cauchi, 2009 (16 years) (11) | 1.25 | 1.17 | 1.33 |
| Zobel, 2009 (13) | 1.28 | 1.19 | 1.38 |
| Cheung, 2010 (16) | 1.24 | 1.17 | 1.33 |
| Shi, 2010 (17) | 1.24 | 1.16 | 1.32 |
| Huang, 2011 (18) | 1.23 | 1.16 | 1.31 |
| Rouskas, 2011 (19) | 1.26 | 1.18 | 1.34 |
| Beckers, 2011 (20) | 1.25 | 1.17 | 1.34 |
| Thomsen, 2012 (21) | 1.28 | 1.19 | 1.37 |
| Tao, 2012 (22) | 1.27 | 1.18 | 1.37 |
| Wu, 2010 (24) | 1.25 | 1.16 | 1.33 |

OR, odds ratio; CI, confidence interval
