# Supplementary material for: Association between Common Polymorphism near the MC4R Gene and Obesity Risk: A Systematic Review and Meta-Analysis
Source: PLoS One. 2012 Sep 25;7(9):e45731. doi: 10.1371/journal.pone.0045731 (PMC3458070; doi:10.1371/journal.pone.0045731)
Supplement: Table S5 — Sensitivity analysis under a recessive model. (DOC) [file pone.0045731.s005.doc]

**Supplementary table 5: Sensitivity analysis under a recessive model**

| **Study omitted** | **OR** | **95% CI** | |
| --- | --- | --- | --- |
| Hotta, 2009 (9) | 1.46 | 1.25 | 1.70 |
| Tabara, 2009 (10) | 1.43 | 1.23 | 1.67 |
| Cauchi, 2009(adult) (11) | 1.40 | 1.21 | 1.63 |
| Cauchi, 2009 (16 years) (11) | 1.43 | 1.23 | 1.65 |
| Zobel, 2009 (13) | 1.47 | 1.24 | 1.74 |
| Cheung, 2010 (16) | 1.40 | 1.21 | 1.62 |
| Shi, 2010 (17) | 1.38 | 1.20 | 1.60 |
| Huang, 2011 (18) | 1.41 | 1.21 | 1.64 |
| Rouskas, 2011 (19) | 1.41 | 1.23 | 1.63 |
| Beckers, 2011 (20) | 1.38 | 1.20 | 1.59 |
| Thomsen,2012(21) | 1.47 | 1.28 | 1.68 |
| Tao,2012(22) | 1.42 | 1.22 | 1.66 |
| Wu, 2010 (24) | 1.36 | 1.18 | 1.55 |

OR, Odds ratio; CI, confidence interval
