# Supplementary figures and images for: Association between Common Polymorphism near the MC4R Gene and Obesity Risk: A Systematic Review and Meta-Analysis
Source: PLoS One. 2012 Sep 25;7(9):e45731. doi: 10.1371/journal.pone.0045731 (PMC3458070; doi:10.1371/journal.pone.0045731)

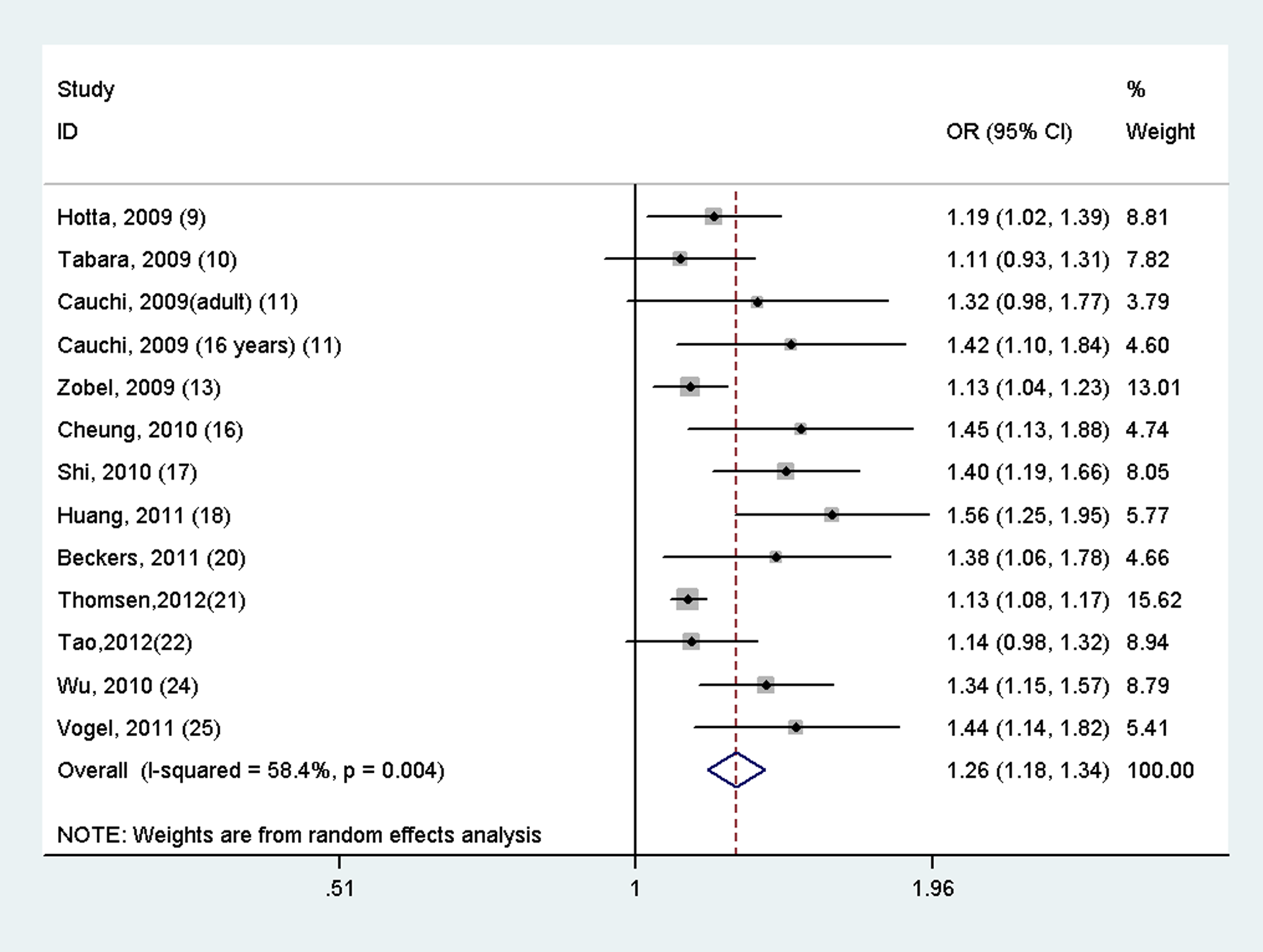

Supplement: Figure S1 — Meta-analysis of the association between rs17782313 polymorphism (or proxy) near the MC4R gene and obesity risk under a dominant genetic mod. (TIF) [file pone.0045731.s007.tif]

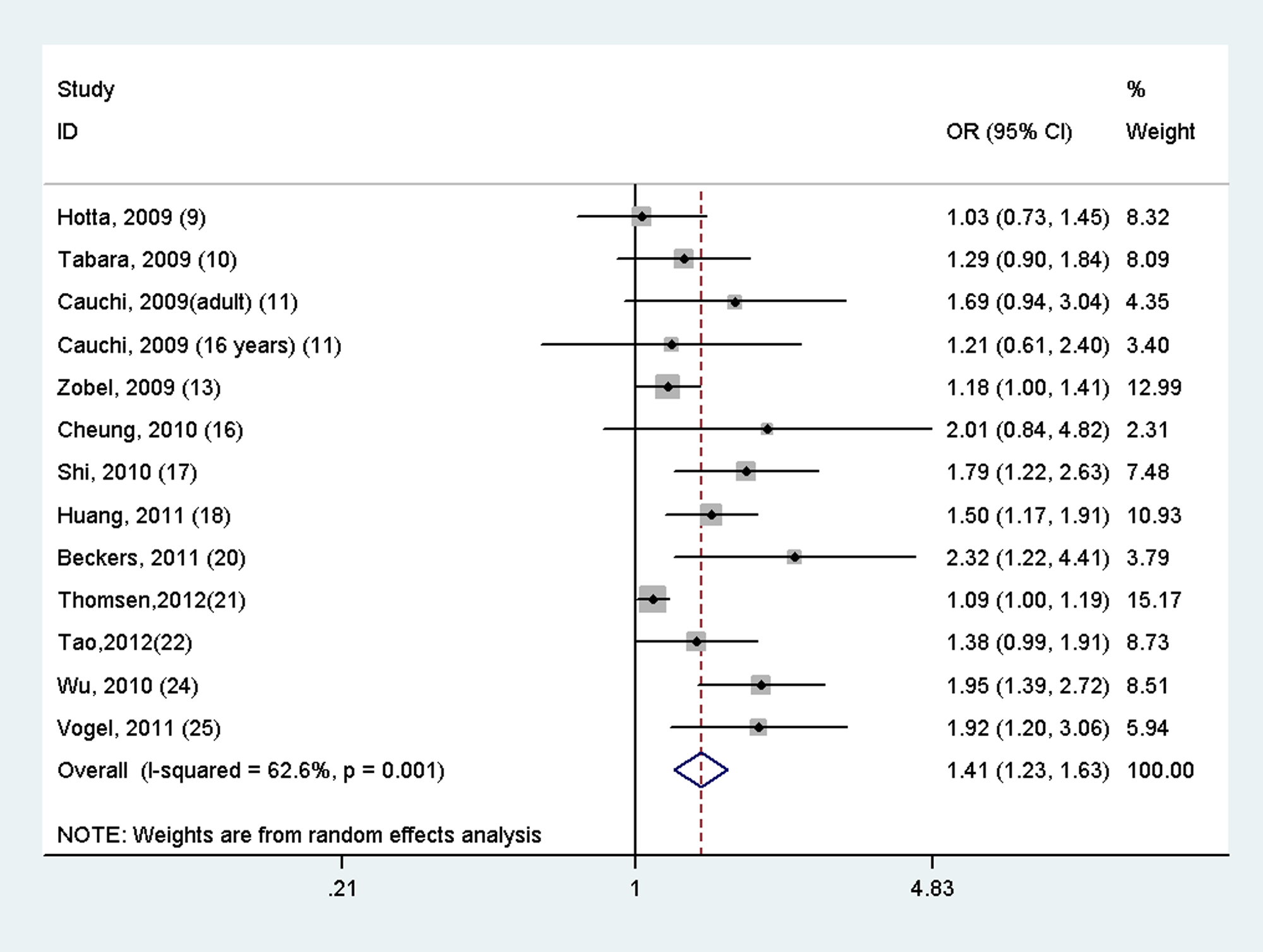

Supplement: Figure S2 — Meta-analysis of the association between rs17782313 polymorphism (or proxy) near the MC4R gene and obesity risk under a recessive genetic model. (TIF) [file pone.0045731.s008.tif]

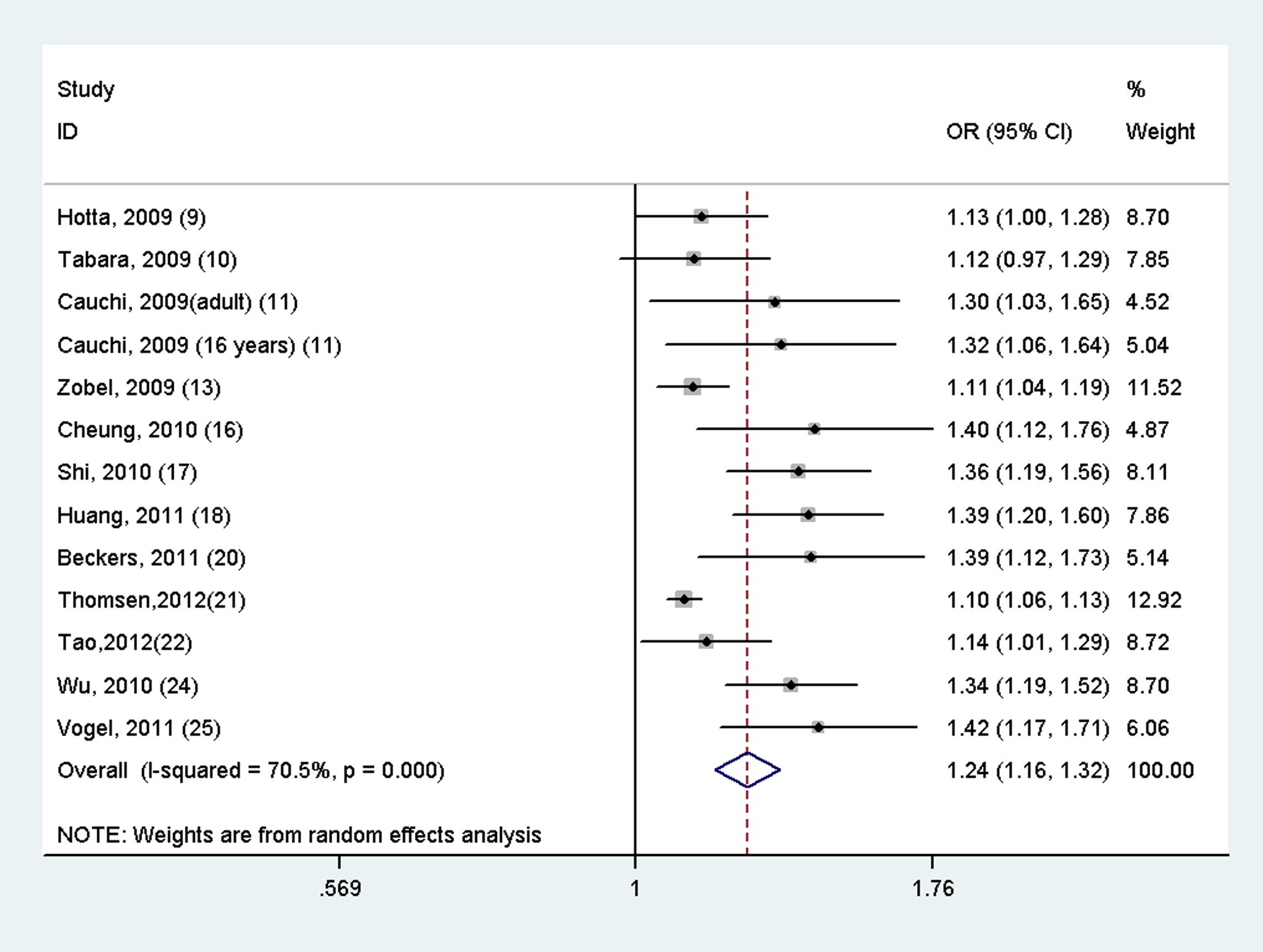

Supplement: Figure S3 — Meta-analysis of the association between rs17782313 polymorphism (or proxy) near the MC4R gene and obesity risk under a allelic genetic model. (TIF) [file pone.0045731.s009.tif]
